# Solid Polymer Electrolytes Based on Gellan Gum and Ionic Liquid for Sustainable Electrochromic Devices

**DOI:** 10.1021/acsami.2c01658

**Published:** 2022-03-24

**Authors:** Raquel Alves, Arkaitz Fidalgo-Marijuan, Lia Campos-Arias, Renato Gonçalves, Maria Manuela Silva, Francisco Javier del Campo, Carlos M. Costa, Senentxu Lanceros-Mendez

**Affiliations:** † Physics Centre of Minho and Porto Universities (CF-UM-UP), University of Minho, 4710-057 Braga, Portugal; ‡ BCMaterials, Basque Center for Materials, Applications and Nanostructures, UPV/EHU Science Park, 48940 Leioa, Spain; § Department of Organic and Inorganic Chemistry, University of the Basque Country (UPV/EHU), 01006 Vitoria-Gasteiz, Spain; ∥ Center of Chemistry, University of Minho, 4710-057 Braga, Portugal; ⊥ Ikerbasque, Basque Foundation for Science, 48009 Bilbao, Spain; # Institute of Science and Innovation for Bio-Sustainability (IB-S), University of Minho, 4710-057 Braga, Portugal; △ Laboratory of Physics for Materials and Emergent Technologies, LapMET, University of Minho, Braga 4710-057, Portugal

**Keywords:** solid polymer electrolytes, gellan gum, ionic liquid, electrochromic devices, sustainability

## Abstract

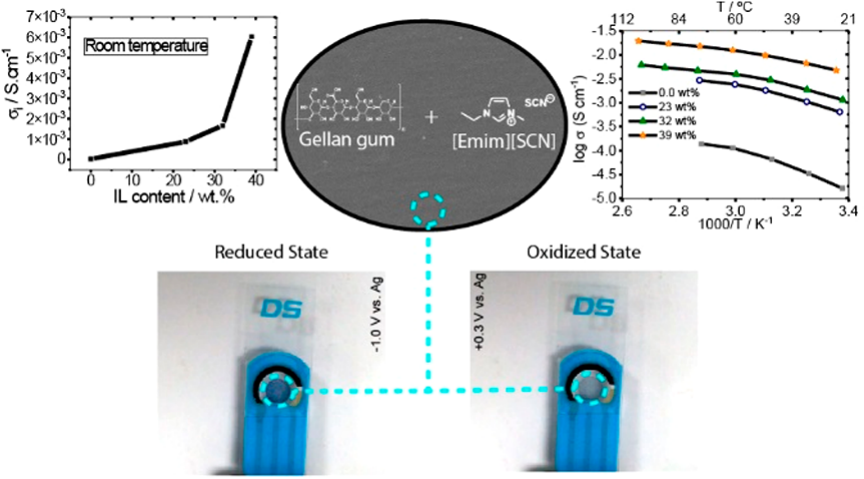

Materials sustainability is becoming increasingly relevant in every developed technology and, consequently, environmentally friendly solid polymer electrolytes (SPEs) based on gellan gum and different quantities of ionic liquid (IL) 1-ethyl-3-methyl-imidazolium-thiocyanate [Emim][SCN] have been prepared and applied in electrochromic devices (ECDs). The addition of the IL does not affect the crystalline phase of gellan gum, and the samples show a compact morphology, surface uniformity, no phase separation, and good distribution of the IL within the carrageenan matrix. The developed SPE are thermally stable up to ∼100 °C and show suitable mechanical properties. The most concentrated sample (39 wt % IL content) reaches a maximum ionic conductivity value of 6.0 × 10^–3^ S cm^–1^ and 1.8 × 10^–2^ S cm^–1^ at 30 and 90 °C, respectively. The electrochromic device (ECD) was fabricated with poly(3,4-ethylenedioxythiophene) polystyrenesulfonate (PEDOT:PSS) as working electrode and the developed SPE was compared with an aqueous 0.1 M KNO_3_ solution. The electrochromic performance of the electrolyte was assessed in terms of spectroelectrochemistry, demonstrating a fully flexible ECD operating at voltages below 1.0 V. This novel electrolyte opens the door to the preparation of high performance sustainable ECD.

## 1 Introduction

Solid polymer electrolytes (SPEs) are being increasingly studied as promising materials to replace inorganic electrolytes and liquid crystals in a variety of electrochemical devices, including batteries, supercapacitors, fuel-cells, and electrochromic devices.^1,2^ SPEs are typically based on a salt within a polymer matrix, forming an ionically conducting solid solution.^3^ Among various investigated systems relying on polymers such as fluoropolymers (poly(tetrafluoroethylene) – PTFE and poly(vinylidene fluoride) – PVDF), those based on natural polymers are getting special attention. The interest in natural polymers results from their specific properties as well as from the fact that, being naturally available and low cost materials, their use strongly reduces the environmental impact of materials and devices. In this context, gellan gum polymer is a deacetylated anionic polysaccharide resulting from a fermentation product of *Pseudomonas elodea* culture.^4^ It contains one −COOH group and two −CH_2_OH groups per repeating unit, which allows the formation of complexes with salts, leading to an increase in ionic conductivity.^5^

The development of novel materials based on proteins and polysaccharides has received increasing attention in recent years, and the challenge is to improve their functional characteristics and, in particular, their inherently low ionic conductivity.^6^

Solid polymer electrolyte ionic conductivity is typically related to the amorphous phase and the incorporation of suitable plasticizers into SPE is a successful approach to increase the amorphous nature.^7^ Further, the ionic conductivity can be enhanced by using a polymer matrix with high dielectric constant, which leads to an improvement in ions dissociation, and low molecular weight, to increase their mobility.^8^ It has been reported that the use of plasticizers can increase the ionic conductivity in 1 or 2 orders of magnitude and decrease the glass transition temperature, *T*_g_, by 40 °C.^9^ In particular, glycerol (C_3_H_8_O_3_) is a nontoxic compound based on a multihydroxyl moiety structure with high boiling (290 °C) and low melting (18 °C) temperatures, and high dielectric constant (42.5).^10,11^ These properties make it interesting for the preparation of electrolytes, as it overcomes the vaporization and solidification process at room temperature, enhances salt dissociation, and decreases polymer–polymer interactions.^12^ It has been widely used in the preparation of electrolytes together with poly(vinyl chloride) (PVC) doped with NH_4_SCN salt,^13^ poly(vinyl alcohol) (PVA): NH_4_SCN: Cd(II)-complex,^14^ carboxymethyl cellulose–NH_4_Br system,^15^ and *Bombyx mori* silk fibroin (SF) hybridized with the ionic liquid 1-butyl-3-methylimidazolium hexafluorophosphate, [Bmim]PF_6_, biopolymer electrolyte,^16^ among others

Ionic liquids (ILs) are molten salts with bulky and asymmetric organic and inorganic cations and anions with highly delocalized charges,^2^ and have been also increasingly used for the development of SPEs. They are nonflammable an present high ionic conductivity, nonvolatility, negligible vapor pressure, excellent thermal and chemical stabilities, wide electrochemical potential window, ability to solubilize both inorganic and organic compounds, are liquid in a wide temperature range, and show low melting temperatures (<100 °C), which make them promising materials for a wide variety of applications.^17^

In particular, IL have been used for the development of SPE based on 1-ethyl-3-methylimidazolium bis(trifluoromethylsulfonyl)imide, [EMIM][TFSI],^18^ 1-butyl-3-methylim-idazolium bis(trifuoromethanesulfonyl)imide, [BMIM][TFSI],^19^ 1-ethyl-3-methylimidazo-lium tetrafluoroborate, [EMIM][BF_4_],^20^ 3-methy-1-propylimi-dazolium bis(trifluoromethysulfony) imide, [PMIM][TFSI],^21^ N-*n*-butyl-*N*-methylpyrrolidinium bis(trifluoromethanesulfonyl)imide, [Pyr14][TFSI],^22^ among others. These ILs have been used to increases the ionic conductivity of SPEs, based on its high conductivity, being, as an example, to 10^–3^ S cm^–1^ at 80 °C values for N-*n*-butyl-*N*-methylpyrrolidinium bis(trifluoromethanesulfonyl)imide, [Pyr14][TFSI].^22^ SPEs with moderate conductivity have been used in the development of electrochromic devices for nearly 20 years.^23^ Even with adequate results, the challenge of SPE for this application is to develop materials with high ionic conductivity between 1 × 10^–3^ and 1 × 10^–4^ S cm^–1^, zero electronic conductivity, good mechanical flexibility, high transparency and high thermal stability.^24^

Poly(3,4-ethylenedioxythiophene):polystyrenesulfonate, PEDOT:PSS is a widely adopted conducting polymer for applications as transparent conductor in displays, and as gate material in organic transistors.^23^ In addition to these main uses, poly(3,4-ethylenedioxythiophene) (PEDOT) is an electrochrome that presents a high cathodic coloration efficiency.^25^ The electrochromism of PEDOT makes it particularly interesting in the development of displays as it can act both as conductor and electrochrome, simplifying device construction.^26^

Once most SPEs are based on synthetic polymers and many of them on fluoropolymers, the novelty of this work is the demonstration of the suitability of a novel eco-friendly SPEs based on gellan gum containing 1-ethyl-3-methyl-imidazolium-thiocyanate ([Emim][SCN]) ionic liquid for the development of electrochromic devices, devices that can be applied in displays, sensors, smart glass, and mirrors, as well as in smart labels and smart packaging.^23,24^ The samples were synthesized and characterized and the SPE were used for the development of a coplanar display that does not involve additional transparent electrodes.

## 2 Experimental Section

### 2.1 Materials

Gellan gum polymer and glycerol C_3_H_8_O_3_ (99.5%) were acquired from Sigma-Aldrich and HiMedia, respectively.

**Scheme 1:**
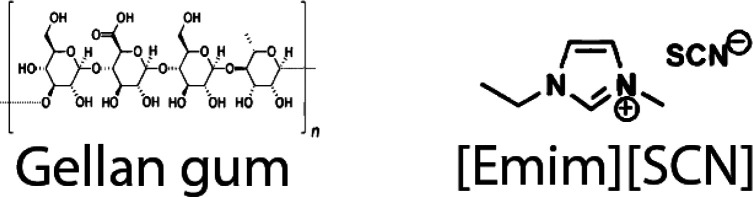
Chemical Structure of the Gellan Gum and the IL [Emim][SCN]

The ionic liquid used in this work was 1-Ethyl-3-methyl-imidazolium-thiocyanate (C_7_H_11_N_3_S–[Emim][SCN]) with high conductivity value (17.8 mS cm^–1^), melting temperature of −6 °C, thermal degradation at 265 °C, and viscosity of 24.7 cP were purchased from Iolitec (Heilbronn, Germany) with a purity of >98%. Scheme 1 shows the chemical structure of the polymer and the IL used in this work.

### 2.2 Solid Polymer Electrolytes Preparation

The solid polymer electrolytes were obtained by solvent casting according to the procedure presented in ref (27). Samples were prepared by dispersion of 0.25 g of gellan gum (Gelzan, Sigma-Aldrich) in 25 mL of ultrapure water (Milli-Q, Gradient A10 Water Purification System), and heated at 60 °C under magnetic stirring for dissolution of the carrageenan polymer. After this, 0.25 g of glycerol (HiMedia, 99.5%) to increase flexibility, and different quantities of ILs (23, 32, and 39 wt %) were added under magnetic stirring. The different ILs quantities were selected in order to improve the ionic conductivity guaranteeing the mechanical stability of the matrix and preventing the gelation of the solution during processing. The resulting solutions were then poured onto Petri dishes, cooled at room temperature until film formation, and subjected to a final drying in an oven at 45 °C during 48 h. The final thickness of all samples is about ∼120 μm.

### 2.3 Samples Characterization

The surface morphology evaluation of the samples was carried out by scanning electron microscopy (SEM) with a Carl Zeiss EVO 40 with an accelerating voltage of 20 kV in samples coated with a conductive gold layer (Polaron, model SC502). Energy dispersive spectroscopy (EDS) was carried out in order to identify different elements with the EDX–Oxford Instruments apparatus at a voltage of 10 kV.

Attenuated total reflection-Fourier transform infrared (ATR-FTIR) spectroscopy measurements were carried out with a Jasco FT/IR-6100 equipment in the spectral range between 4000 and 600 cm^–1^ after 64 scans with a resolution of 4 cm^–1^.

X-ray diffraction (XRD) measurements were obtained in the range of 5 < 2θ < 70° with a step size of 0.05° and an exposure of 10 s per step using a Philips X’Pert PRO diffractometer with CuKα radiation (λ = 1.5406 Å).

The thermal stability of the samples was assessed by thermogravimetric analysis, TGA, (NETZSCH STA 449F3) in heating scans from 20 to 800 °C at 5 °C min^–1^ under an argon atmosphere, in a crucible (±10 mg). DSC measurements were carried out between 20 and 200 °C at a heating rate of 10 °C min^–1^ in a PerkinElmer DSC 6000 equipment, under a nitrogen atmosphere.

The ionic conductivity value was determined through the electrochemical impedance spectroscopy using the gellan gum based electrolytes between two ion-blocking gold electrodes (10 mm diameter).^27^ The measurements were carried out using an Autolab PGSTAT-12 (Eco Chemie) at frequencies between 0.5 MHz and 0.5 Hz and temperatures from 20 to 100 °C.

### 2.4 Electrochromic Device

We manually applied 10 × 10 mm pieces of the solid polymer electrolyte to cover the working, auxiliary and pseudoreference electrodes of commercial screen-printed PEDOT and carbon electrodes reference P10 and DRP-110, respectively (DropSens, ES). A SPELEC UV–vis spectroelectrochemistry apparatus (Metrohm-Dropsens, ES; DropView SPELEC software) was used for the spectroelectrochemical experiments. The transmission cell (TRANSCELL; DropSens, ES) was used in the experiments. Unless otherwise stated, all potentials are reported versus Ag. In all cases, the open circuit potential (OCP) was determined first to ensure that all subsequent experiments started at a zero-current level.

## 3 Results and Discussion

### 3.1 Morphology, Structure, and Chemical Interaction

**Figure 1:**
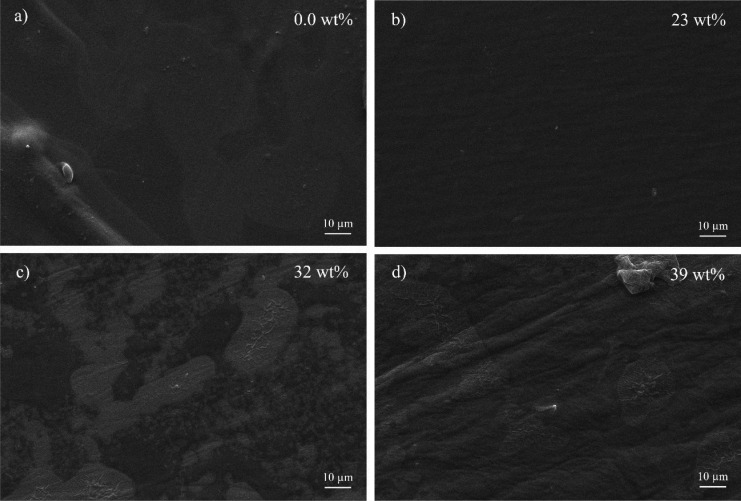
SEM images of a) gellan gum matrix and gellan gum-based electrolytes with b) 23, c) 32 and d) 39 wt % of [Emim][SCN].

In order to assess the effect of the [Emim][SCN] inclusion in the morphology of the gellan gum samples, SEM and EDS images were recorded (Figure 1). Figure 1a) shows the surface SEM image for the gellan gum matrix, showing a surface uniformity and excellent homogeneity without phase separation. The same behavior is observed in the sample with low IL amount, namely, 23 wt % (Figure 1b). Increasing IL content to 32 and 39 wt % leads to some phase separation, probably related to IL located on the surface of the sample (Figure 1c and d) as observed in a previous work.^27^

**Figure 2:**
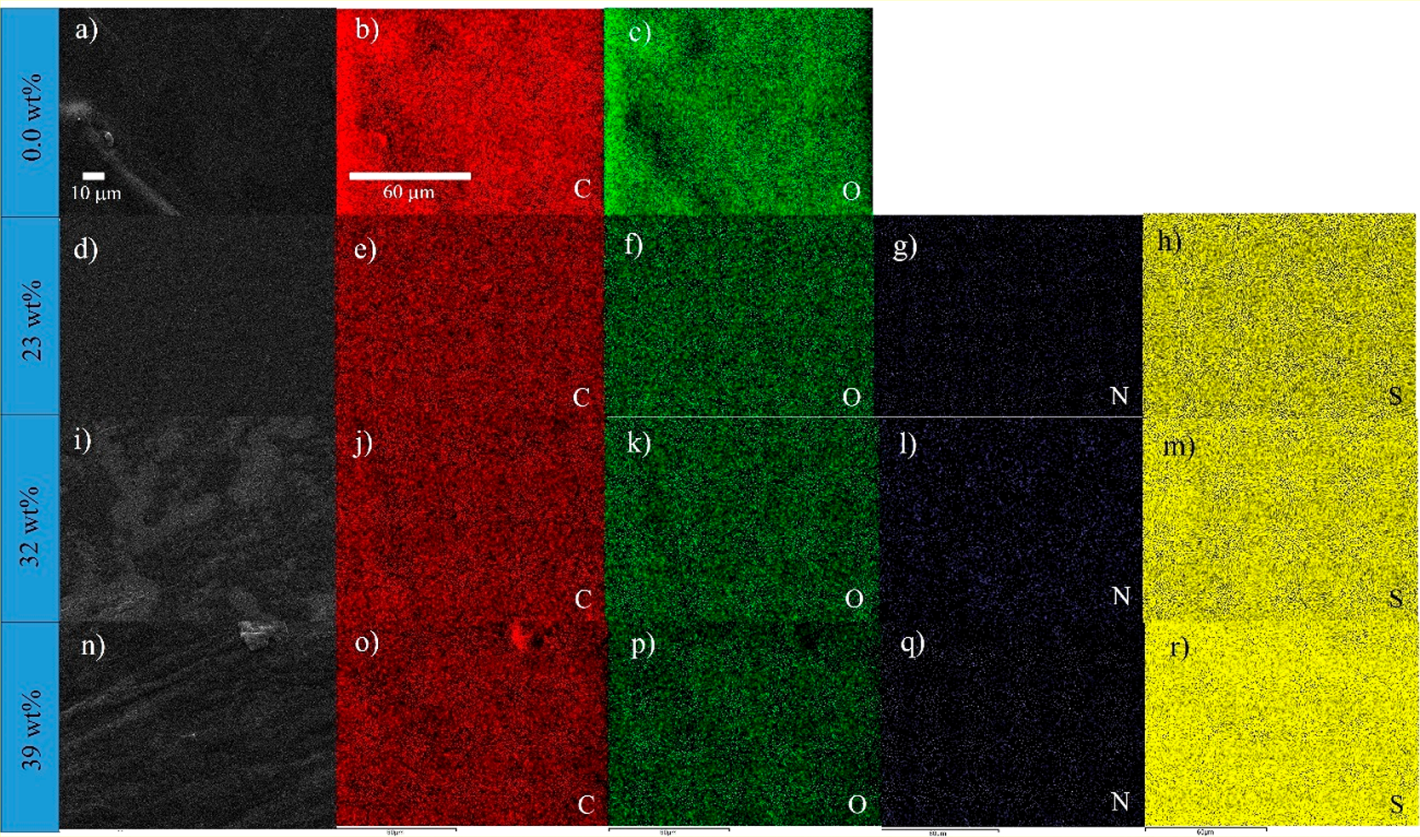
SEM images of gellan gum and the SPEs based on gellan gum and [Emim][SCN], and the corresponding EDS mapping images for C (carbon, red, 2b, 2e, 2j, and 2o), O (oxygen, green, 2c, 2f, 2k, and 2p), N (nitrogen, violet, 2g, 2l, 2q), and S (sulfur, yellow, 2h, 2m, 2r) atoms.

The EDS mapping images of the gellan gum matrix and the corresponding electrolytes with [Emim][SCN] (Figure 2) confirm the homogeneous distribution of the different elements (carbon and oxygen) within the samples. Nitrogen and sulfur atoms are related to the IL structure showing also a homogeneous distribution of those atoms in the matrix surface.

**Figure 3:**
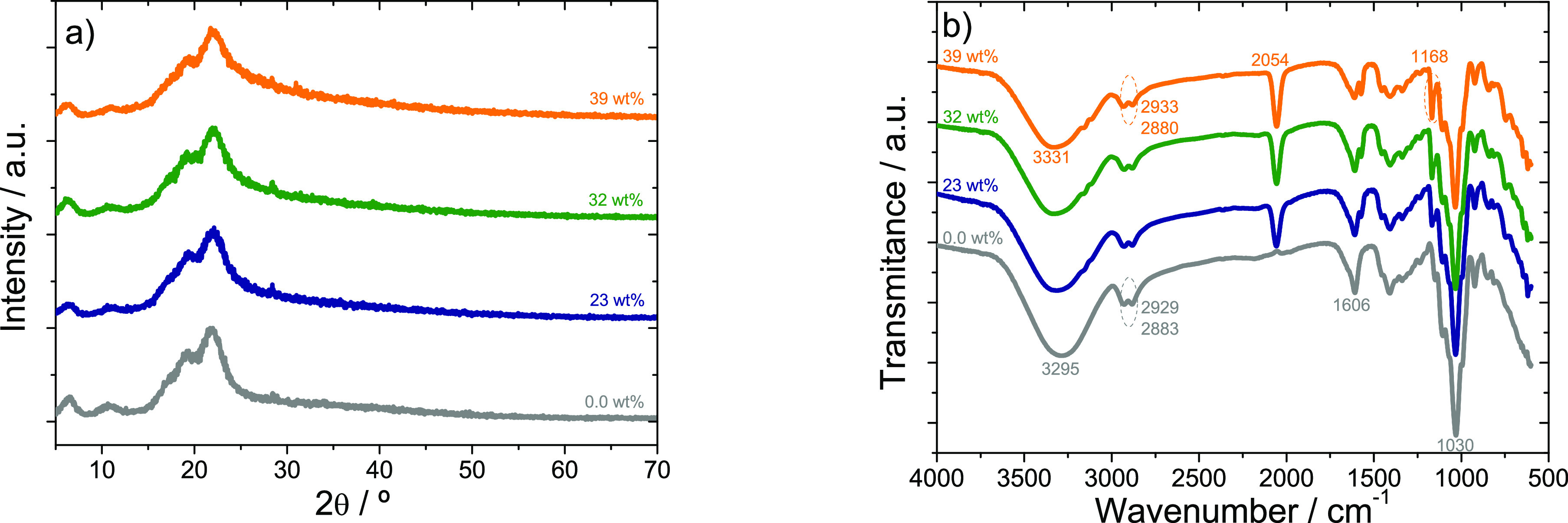
(a) XRD patterns and (b) FTIR spectra of gellan gum matrix and gellan gum based hybrid electrolytes with [Emim][SCN].

The XRD patterns of the polymer electrolytes based on gellan gum are presented in Figure 3a), together with the one of the pristine polymer, which is characterized by intense peaks at 2θ = 6°, 10°, 19°, and 22°.^28^

In fact, for the pristine gellan gum matrix, diffraction peaks are observed at 2θ = 6°, 11°, 19°, and 22°, as reported in the literature.^28^ The presence of these diffraction peaks confirm the crystalline domains in its structure.^27^ The XRD of the hybrid samples also presented peaks at 2θ ∼ 6°, 11°, 19°, and 22°, with a broadening in the diffraction peaks revealing a decrease in crystallinity of the hybrid samples. This behavior is related to the IL acting as defect during the crystallization process.^27^ Furthermore, no other peaks are observed in the electrolytes containing [Emim][SCN].

The FTIR spectra of the different samples have been used to analyze the possible chemical interactions between the polymer matrix and the [Emim][SCN]. Figure 3b) shows the FTIR spectra of pristine gellan gum and gellan gum hybrid electrolytes with different IL contents. The gellan gum matrix shows a broad band at 3295 cm^–1^ corresponding to the O–H stretching vibration of the hydroxyl groups of polysaccharide molecules.^29^ This broad peak shifts to 3310 cm^–1^, 3330 cm^–1^, and 3331 cm^–1^ for the electrolytes with 23, 32, and 39 wt % IL content, respectively, indicating the interaction of the IL with the polymer matrix. The bands in the range from 2883 to 2929 cm^–1^ in gellan gum are ascribed to CH_3_ and CH_2_ stretching vibration groups.^30^ These bands shift to 2880 and 2933 cm^–1^, respectively, in the electrolyte with 39 wt %. A band at 2054 cm^–1^, which does not exist in the spectrum of gellan gum, is related to the IL anion [SCN^–^].^31^ The band observed at 1606 cm^–1^ is probably due to the glycosidic link in gellan gum.^32^ This peak is also observed in samples containing IL, whose intensity decreases with increasing [Emim][SCN] amount. The band at 1409 cm^–1^ in gellan gum is related to C–H bending^33^ and the intensity of this band decreases with increasing IL content. The vibrational peak at 1149 cm^–1^ for gellan gum and the one at 1168 cm^–1^ for gellan gum hybrid electrolytes is related to C–C stretching vibration. The peak at 1030 cm^–1^ observed in the gellan gum matrix corresponds to C–O stretching vibration^4^ and the same peak also appears in the samples containing [Emim][SCN].

### 3.2 Thermal Properties

**Figure 4:**
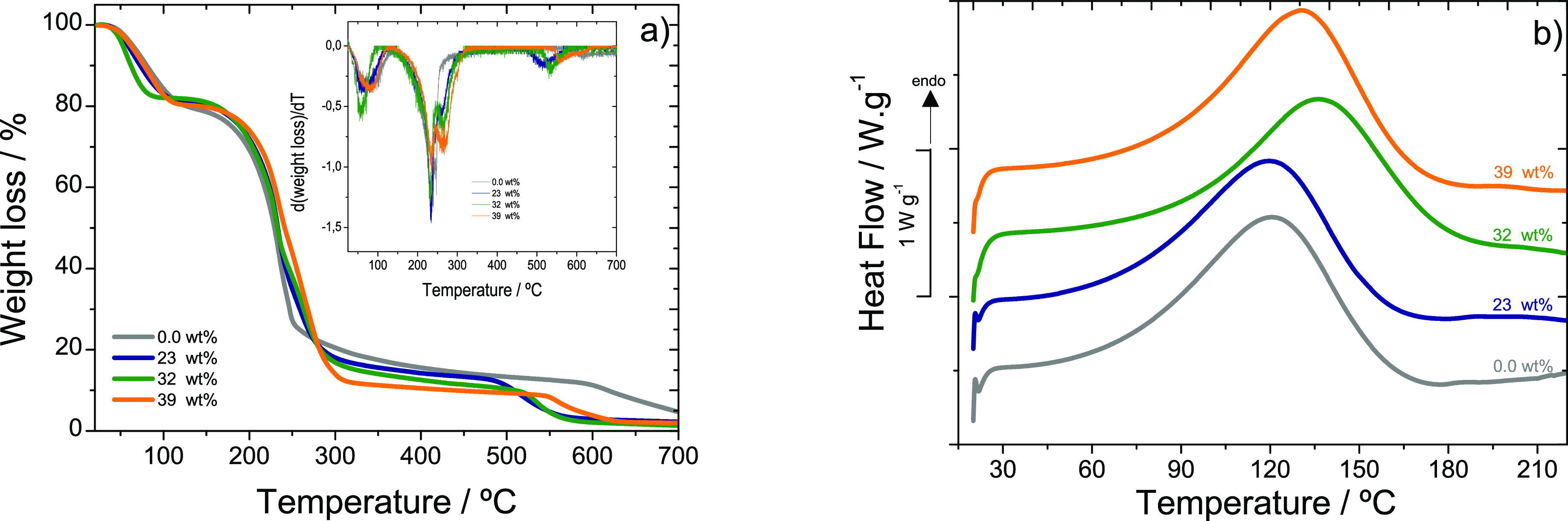
(a) TGA (insert: DTG) and (b) DSC thermograms of gellan gum and gellan gum-based hybrid electrolytes with [Emim][SCN].

The thermal stability of the gellan gum-based samples was evaluated by thermogravimetric (TGA) analysis. Figure 4a) shows the TGA thermograms of the gellan gum matrix and gellan gum-based electrolytes with different IL contents (from 23 to 39 wt %), where different degradation steps are detected as also shown in the derivative thermogravimetry (DTG) curves (insert Figure 4a). All samples show a weight loss of 18–20% that starts at about 40–50 °C, which is related to the water loss in the samples. The gellan gum matrix shows a very accentuated degradation step at 210 °C with weight loss of 63%. The second step presents a weight loss of 13% and the final residue of the sample is 5%. In the case of the hybrid samples, the main degradation occurs between 206 and 212 °C with a weight loss between 65 and 69%, demonstrating that the presence of the IL does not expressively change the degradation process of the polymer matrix. The second degradation step starts between 490 and 546 °C resulting in 10–15% of weight loss and a final residue between 1.28 and 2.24%. This weight loss occurs at a lower temperature, when compared with the pristine gellan matrix, which is ascribed to the different interactions of the polymer backbone with the IL. A report on gellan gum-LiCF_3_SO_3_ electrolytes also showed that the samples are stable up to 234 °C,^33^ with an initial weight loss related to loss of water followed by two degradation steps.

Figure 4b) shows the DSC curves of gellan gum matrix and hybrid samples with [Emim][SCN]. Regardless of the sample type, a sharp peak centered at 120 and 130 °C is observed, attributed to a melting of the crystalline region of the gellan gum matrix.^30^ The melting temperatures (*T*_m_) of the hybrid samples shift to higher temperatures in comparison with pristine gellan gum due to the interaction between the IL and the polymer, effect that depends on the presence of the IL within the polymer matrix but does not depend on the IL content. The *T*_m_ of pristine gellan gum is around 116 °C^32^ an in gellan-LiI based samples a peak at about 125 °C was observed, which is ascribed to the melting of the gellan gum crystalline phase.^30^ In the present study, *T*_m_ for the gellan gum matrix is 121 °C, and in the hybrid samples, this value increases for the most concentrated samples reaching a maximum value of 136 °C.

### 3.3 Mechanical and Impedance Analysis

**Figure 5:**
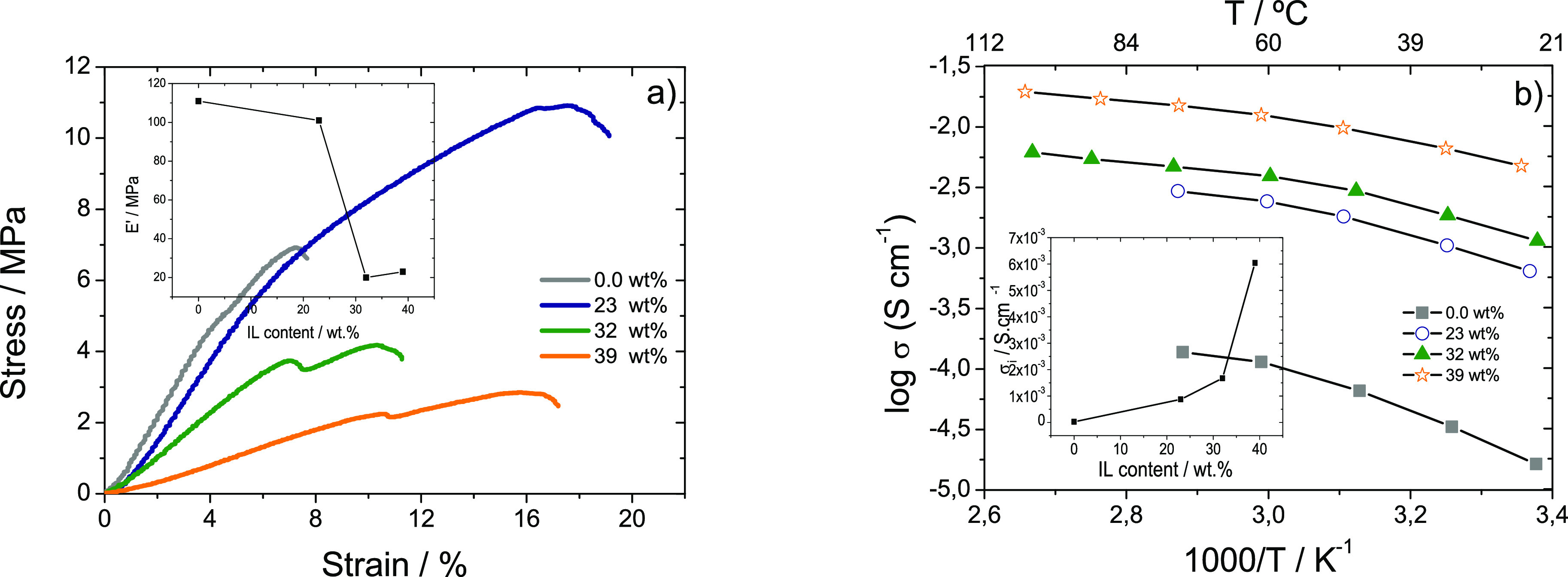
(a) Stress–strain curves. The inset shows the Young modulus as a function of IL content in the polymer matrix. (b) ionic conductivities as a function of temperature for the gellan gum matrix and gellan gum electrolytes containing [Emim][SCN]. The inset shows the ionic conductivity of the samples as a function of IL content.

The stress–strain mechanical characteristic curves of the gellan gum matrix and gellan gum-based hybrid electrolytes with different IL content are shown in Figure 5a. Regardless of the samples, Figure 5a) shows the typical mechanical curve of the gellan gum matrix^34^ characterized by an elastic and a plastic region. The Young modulus of the samples was determined by the tangent method at 3% of deformation in the elastic region^35^ and it is observed to decrease with increasing IL content, that is, for gellan gum matrix and gellan gum electrolyte with the highest IL amount, the Young modulus is 111 and 23 MPa, respectively (insert in Figure 5a).

It is also observed that the tensile strength decreases linearly with increasing IL content except for the sample with 23 wt % IL. This effect is due to the plasticizing effect of the IL, which increases film flexibility and reduces its brittleness.^36^

The ionic conductivity as a function of temperature for the gellan gum matrix and the corresponding samples doped with [Emim][SCN] are presented in Figure 5b). The conductivity depends mainly on three factors: temperature, dopant concentration, and types of cation and anion.^37^Figure 5b shows that the ionic conductivity value increases with increasing temperature, which is ascribed to the formation of free volume and unoccupied spaces for migration of ions, with increases mobility at higher temperature.^38,39^ The electrical conductivity values also increase with increasing IL content in the samples from 2.7 × 10^–5^ S cm^–1^ for the pristine gellan gum matrix up to a maximum value of 6.0 × 10^–3^ S cm^–1^ at 30 °C and 1.8 × 10^–2^ S cm^–1^ at 90 °C for the sample with 39 wt %. This increase is related to the higher number of charge carriers within the polymer matrix and the fact that a higher ionic liquid content promotes the dissociation of the ion transport from the gellan gum chain motion.^16,39^ The ionic conductivity value at room temperature as a function of ionic liquid content is shown in the inset of Figure 5b).

The ionic conductivity obtained at 30 °C is high when compared to those obtained for gelatin and 1-ethyl-3-methylimidazolium acetate (1.2 × 10^–4^ S cm^–1^),^40^ gellan gum with lithium iodide (LiI) (3.8 × 10^–4^ S cm^–1^)^30^ and gellan gum lithium bis(trifluoromethanesulfone)imide (LiTFSI) (2.77 × 10^–4^ S cm^–1^),^41^ applied in electrochromic devices. Regardless of the sample, it is detected that the ionic conductivity value increases as a function of temperature, as higher temperature increases the polymer chain segmental motion, leading to inter and intrachain ion movements that improve the ion hopping mechanism.^2,42^

### 3.4 Electrochromic Device

Considering the mechanical properties and high ionic conductivity value, the gellan gum electrolyte with 39 wt % was selected to develop the electrochromic devices. The accessible potential window was measured by cyclic voltammetry at the carbon electrode and compared to that of a 0.1 M KNO_3_ solution. In line with similar systems also based on ionic liquids,^43,44^ the accessible potential window of this electrolyte was at least 1.0 V wider than the aqueous electrolyte. This makes this system interesting for its use in combination with organic electrochromic systems with redox potentials beyond 1.5 V vs Ag. The material was found to be electrochemically inert between −2.0 V and +2.0 V vs Ag despite the presence of some water in the gellan gum electrolyte.

A 10 × 10 mm 120 μm-thick sample was sandwiched between two 100 μm-thick transparent PET covers, and the transmitted light was measured between 400 and 900 nm. The light spectrum transmitted through the two PET covers without the electrolyte was taken as reference (100% transmittance). A transmittance of 70–74% was found through the electrolyte across the spectral range studied with no particular absorption bands.

The spectroelectrochemical behavior of commercial PEDOT electrodes in an aqueous electrolyte and in contact with the electrolyte was compared. Aqueous electrolyte experiments were performed on 120 μL of solution, and the electrolyte film covering the three screen-printed electrodes was less than 120 μm thick.

**Figure 6:**
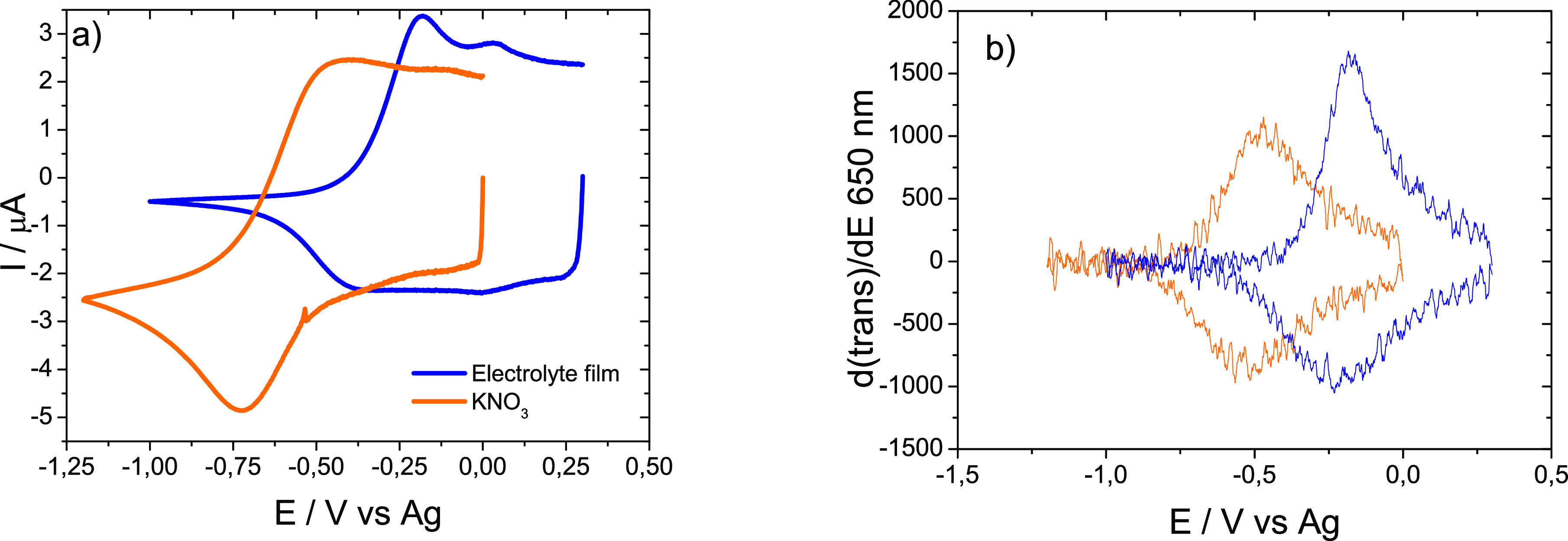
(a) Cyclic voltammograms and (b) voltabsorptograms of a PEDOT electrode in a 0.1 M KNO_3_ solution (orange trace) and in contact with a gellan gum electrolyte film (blue trace).

Figure 6 shows cyclic voltammograms and voltabsorptograms (650 nm) of commercial PEDOT electrodes in both electrolyte systems. These voltammograms were recorded after determining the open circuit potential (OCP) in both systems. The OCP was roughly 300 mV higher in the electrolyte system. This potential difference is due to the presence of SCN^–^ as the IL counterion. Thiocyanate interacts with the Ag pseudoreference electrode, shifting its potential.

**Figure 7:**
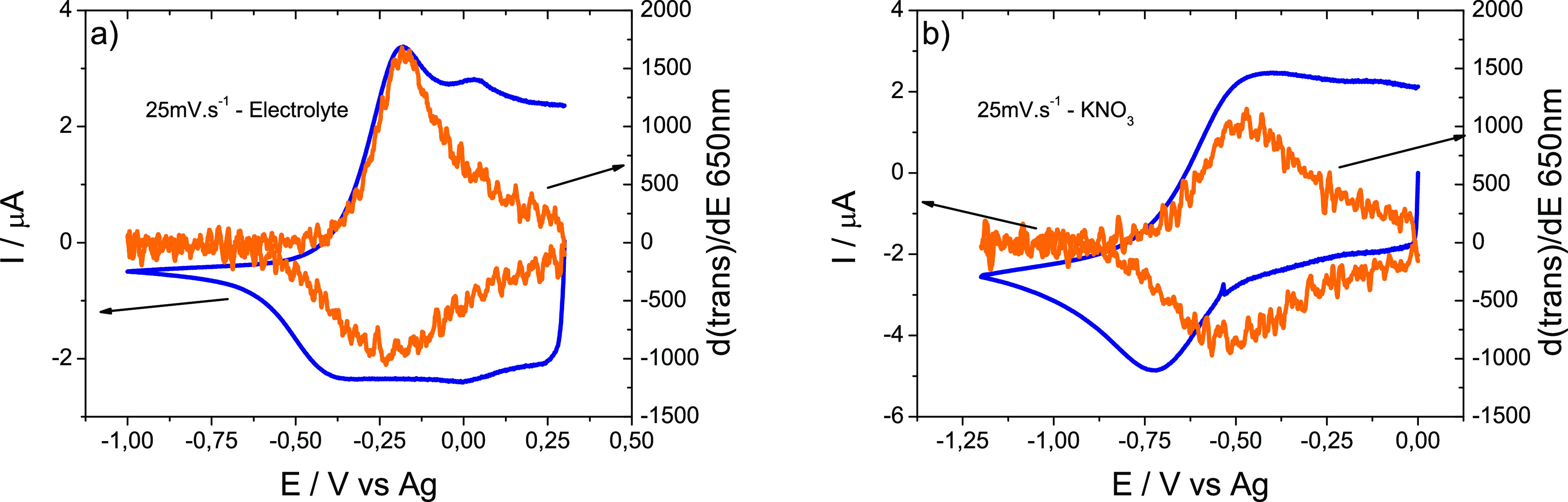
Twenty-five mV s^–1^ cyclic voltammograms and voltabsorptograms recorded at a PEDOT electrode in contact with (a) solid electrolyte film and in (b) 0.1 M KNO_3_ solution. Current (left axis) is depicted in blue while the derivative of transmitted light is colored orange (right axis).

The lower reduction currents and the better definition of the reduction and oxidation peaks at the electrolyte are consistent with this. The voltabsortograms at 640–650 nm show that the signal in the solid electrolyte is more intense than in the aqueous electrolyte, even though the electrochromism of PEDOT is highly reversible in both electrolyte systems. Figure 7 shows that the oxidation peak in the voltammogram has a corresponding spectroscopic signal (PEDOT anodic bleaching). However, the cathodic coloration process does not seem to correspond to any of the reducing currents in the voltammograms. This is because the reduction of dissolved oxygen masked the PEDOT electrode response in both cases. Voltammetric signals are better defined at the solid electrolyte because ion diffusion is slower than in the aqueous electrolyte. These ions are the ions balancing the charge in the PEDOT layer. The PEDOT is not diffusing, it is fixed to the electrode, but its reduction and oxidation also involve the exchange of anions and solvent molecules (conducting redox polymers also undergo swelling and contraction).

**Figure 8:**
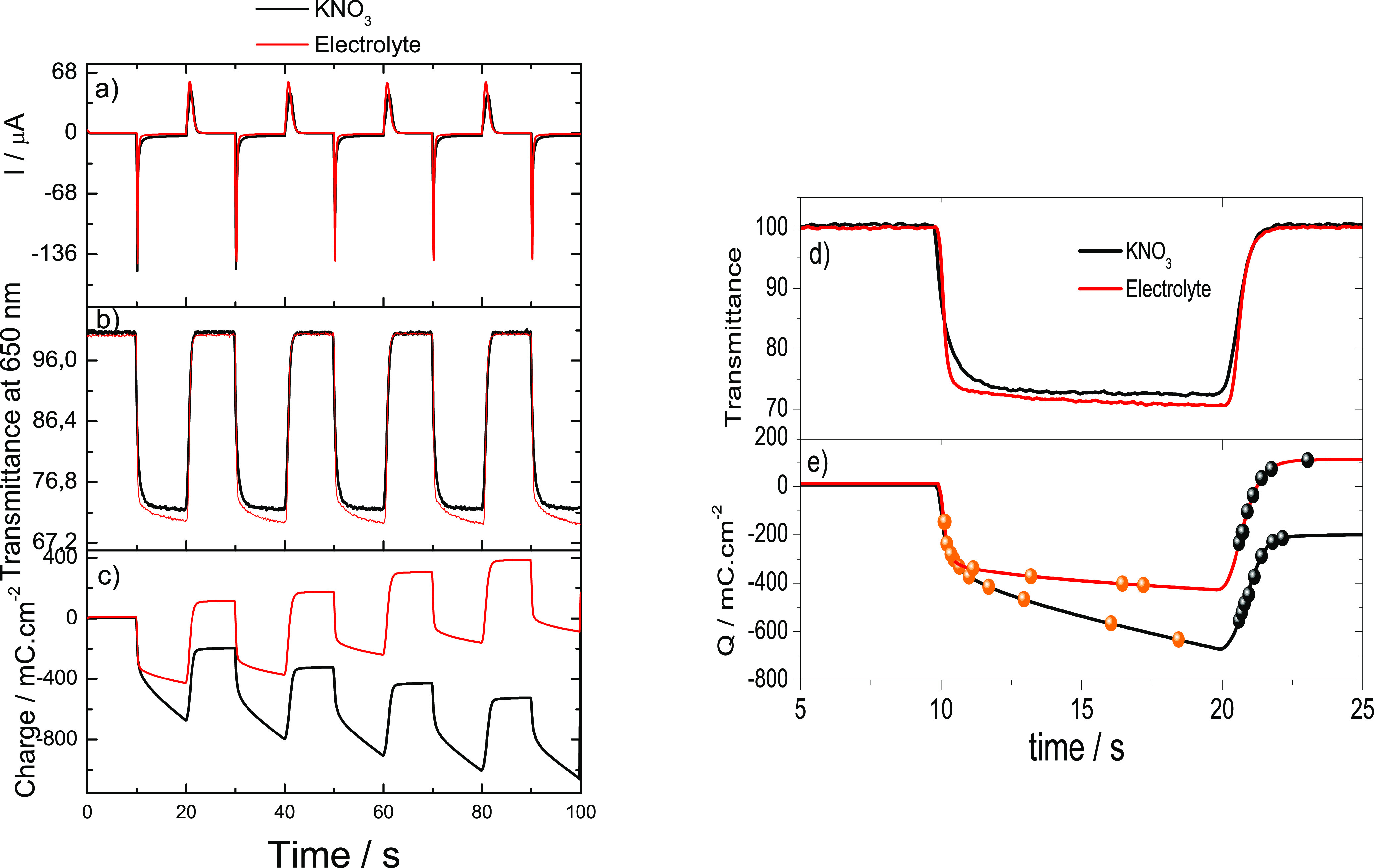
Ten s potential steps between 0.0 and −1.2 V vs Ag at a PEDOT electrode in 0.1 M KNO_3_ and 120 μm-thick electrolyte film: (a) current response, (b) optical electrode transmission at 650 nm, (c) total charge passed, (d) typical transmission change during two consecutive steps, and (e) total charge passed during the step depicted in (d). The dots highlight the time when the electrode was at 50, 60, 70, 80, 90, 95, 98, and 99% of its full contrast.

Transmitted light changes were recorded during a series of potential steps between 0.0 V and −1.2 V and between 0.3 V and −0.9 V in the cases of the aqueous and the solid electrolytes, respectively. The response was partly analyzed according to the methodology described by Reynolds et al.^45,46^Figure 8 show the transient current steps and optical response of PEDOT at the aqueous and solid electrolyte systems.

**Figure 9:**
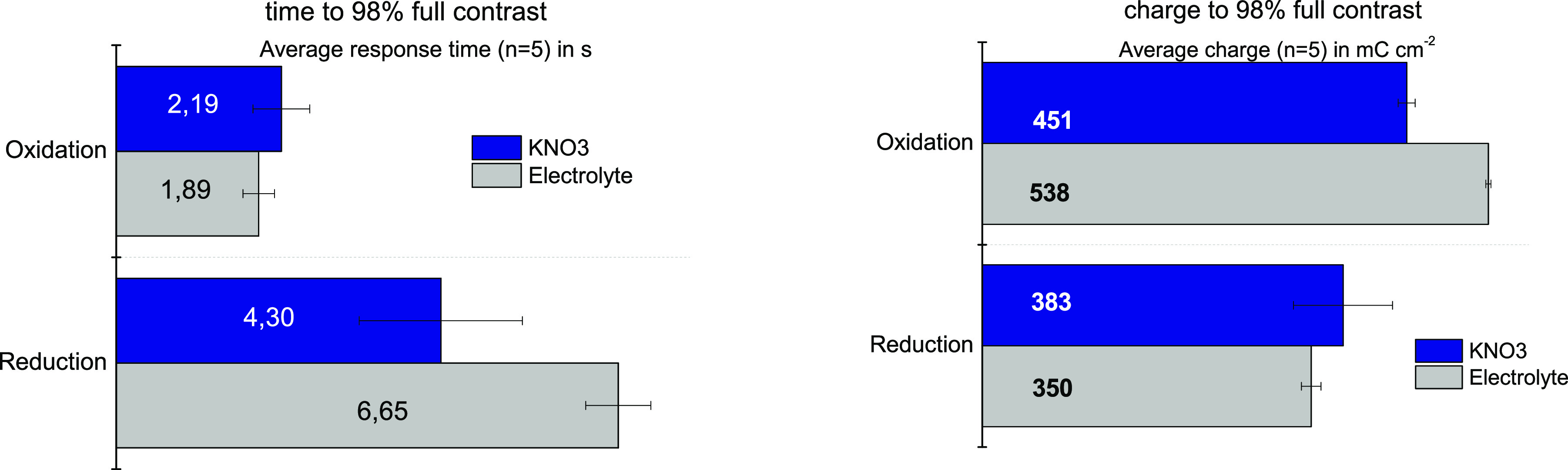
Comparison between the response of a PEDOT electrode in an aqueous electrolyte solution and in contact with the new solid electrolyte for the response time (left) and charge consumed (right).

The data show that the electrochromic response of PEDOT in the solid electrolyte is slower than in KNO_3_ but, on the other hand, the contrast achieved is a tad higher (Figure 9). Indeed, the Δ%T found between the bleached and colored states through the electrolyte is 30.1 ± 0.1% (*n* = 5), whereas in KNO_3_ the maximum Δ%T measured is 28.4 ± 0.2% (*n* = 5). The differences in response are in this case attributed to the ion exchange processes associated with the reduction and oxidation of PEDOT. Electrochromic processes usually involve not only the exchange of electrons, but also of ionic species.^47^ Despite the high ionic conductivity of the solid electrolyte used in this study, ion mobility is still higher in water, thus favoring a faster response (ionic radii for anion is 0.213 nm).^48^ It is interesting to note that the charge seems to be drifting in both cases, albeit in different directions. The case in water is explained by the irreversible reduction of oxygen that occurs together with the reduction of the PEDOT electrode itself. The case of the solid electrolyte, on the other hand, is surprising, because more charge seems to be spent on the anodic processes. One plausible explanation is that this charge stems from ionic exchange processes between the PEDOT and the solid electrolyte.

The switching time for most electrochromic systems as PVDF-HFP with [BMIM][TFSI]^25^ and dry acetonitrile with LiCO_4_^26^ is in the range of a few seconds, in line with the results presented here.

**Figure 10:**
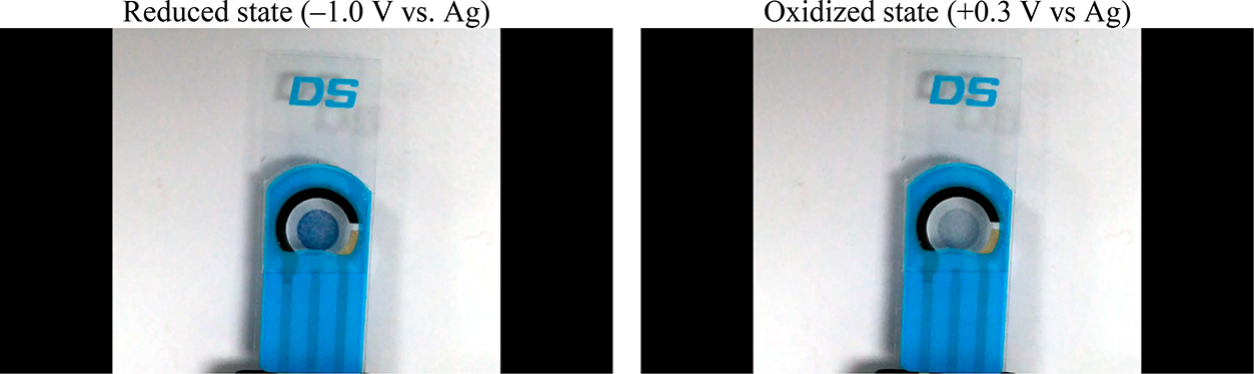
Photographic images of the electrochromic device

Figure 10 shows the photographic images of the electrochromic device with electrolyte in the reduced and oxidized states.

In fact, the use of the developed solid electrolyte has brought about two important advantages: higher color contrast, and easier integration than liquid electrolytes. Further, it is also expected a high stability of the IL based SPE.^49,50^ Liquid electrolytes require tight seals to prevent leaks, whereas solid electrolytes are much more forgiving in terms of assembly and manipulation. The main critical point is ensuring an intimate contact with the electrodes, which can nevertheless be easily achieved by lamination or other means to apply pressure.

## 4 Conclusion

Environmentally friendly solid polymer electrolytes (SPEs) based on gellan gum and containing different amounts of [Emim] [SCN] ionic liquid (IL) have been obtained and applied in electrochromic devices with high performance and improved sustainability. All samples exhibit a compact morphology with no phase separation due to the complete distribution of the ionic liquid in the gellan gum matrix. The thermal properties and crystalline phase of gellan gum are not affected by the ionic liquid addition. Regarding mechanical properties, the Young modulus decreases with increasing the ionic liquid contact from 111 to 23 MPa for gellan gum matrix and gellan gum electrolyte with the highest IL amount (39 wt %), respectively.

At room temperature (30 °C), the ionic conductivity value is 2.7 × 10^–5^ S cm^–1^ for the pristine gellan gum matrix and of 6.0 × 10^–3^ S cm^–1^ and 1.8 × 10^–2^ S cm^–1^ for the sample with higher ionic liquid content (39 wt %) at 30 and 90 °C, respectively.

The electrochromic devices manufactured with this SPE with PEDOT:PSS as a reference electrode operate at voltages below 1 V with a Δ%T between the bleached and colored states of 30.1 ± 0.1% (*n* = 5). Thus, the developed sustainable SPE leads to higher color contrast and easier integration than liquid electrolytes.
